# Synergistic integration of energy harvesters and supercapacitors for enhanced performance

**DOI:** 10.1016/j.heliyon.2025.e42808

**Published:** 2025-02-20

**Authors:** Mariya Aleksandrova, Ivaylo Pandiev

**Affiliations:** a Technical University of Sofia, Dept. of Microelectronics, 8 Kliment Ohridski Blvd, 1756, Sofia, Bulgaria; b Technical University of Sofia, Dept. of Electronics, 8 Kliment Ohridski Blvd, 1756, Sofia, Bulgaria

**Keywords:** Flexible energy harvesters, Piezoelectric generators, Supercapacitors, Wearable power supply

## Abstract

In this paper, it is integrated a piezoelectric energy harvester and a supercapacitor storage device on a flexible substrate with a connection through an innovative alternative current (AC) to direct current (DC) boosting power management system for wearable biosensors’ power supply. Flexible substrates can conform to irregular surfaces or shapes, enabling energy harvesting and storage devices to be integrated into a variety of form factors, including curved or bendable surfaces. Having an integrated energy harvester and storage system ensures a reliable and portable power source, providing power autonomy. The proposed element was layer-by-layer design including silver electrode, polyvinylidene fluoride-trifluoroethylene/multiwall carbon nanotubes, poly(3,4-ethylenedioxythiophene) polystyrene sulfonate: carbon nanotubes, aluminium oxide, graphene and poly(3,4-ethylenedioxythiophene) polystyrene sulfonate: carbon nanotubes (Ag/PVDF-TrFE:MWCNT/PEDOT:PSS:CNT/Al_2_O_3_/Gr/PEDOT:PSS:CNT), prepared by spray coating. A voltage rectifier with a low-pass filter and a direct current to direct current (DC-DC) converter was used as a power management system and intermediate unit between the harvester and storage part of the element. The type of the electronic circuit is voltage-doubler rectifier. It was found that piezoelectric harvester can generates voltage with a magnitude of 2V at loading of 110 g/cm^2^@10 Hz and with the proposed electronic circuit can be determined the workability of the created element during repeated charging and discharging, without introducing interfering changes in the capacity. The behaviour of the supercapacitor part is dependent on the thickness of Al_2_O_3_ and demonstrates more favourable characteristics at the thicker film of 750 nm, where the charging time is short (6s), the voltage ripples are small (±0.50 mV), and the maximum output voltage after charging almost reached the input supply voltage (∼1.94 V output voltage at 2 V input voltage). In addition, it resists up to 15500 cycles and shows a stable retention capacitance of 1.63 mF. The devices retain their capacity at multiple bending (1000) to 93 % and 91 %, according to the aluminium oxide film thickness, which is suitable for wearable devices.

## Highlights

• Obtained thin-film energy harvester and supercapacitor synergistic integration.
• Novel Ag/PVDF-TrFE:MWCNT/PEDOT:PSS:CNT/Al_2_O_3_/Gr/PEDOT:PSS:CNT element is explored.
• High porosity and surface area of Al_2_O_3_ improve the performance of supercapacitor.
• New AC - DC boosting power management system is proposed for wearable power supply.
• Solution for self-sufficient power supply of wearable electronics is proposed.

## 1 Introduction

The integration of energy harvesting and storage technologies has emerged as a promising avenue for advancing the efficiency and functionality of energy systems [1]. Most of the research treating the question with integration of the energy harvesting and energy storage elements have been focused on solar cells and electrochromic batteries for smart windows [2]. Like most of the proposed designs, the focus is on the macro-applications such as smart buildings, or smart homes. For portable devices, the focus is on the solar energy sources because of the DC nature of the output electrical energy, which doesn't require specific power management for the supercapacitor charging [3]. Another scenario involves wireless operation devices like unmanned aerial vehicles (drones, robots), often deployed in remote or hazardous locations, or implantable biosensors with difficult access for maintaining the battery. These devices must be self-sustaining, deriving power from natural sources such as heat, solar, or wind energy, as well as from the human body through piezoelectricity or tribology [4]. While thin-film batteries have shown significant potential, they are not yet fully optimized. Researchers and engineers are continuously working to improve their performance and address various challenges, related to the ions transportation [5,6].

Piezoelectric nanogenerators are the preferable choice for energy harvesting when wearable biomedical electronics are in question [7]. They easily provide the necessary power in the range from a few tens of microwatts for temperature and cardio sensors to a few hundred microwatts for blood glucose and oxygen meters [8]. Traditional piezoelectric materials like ceramics can be brittle and less flexible, limiting their applications in flexible electronics. By exploring piezoelectric polymer composites, a more flexible and lightweight material solution can be achieved. These composites can combine the piezoelectric properties of certain polymers with other materials to enhance flexibility and stretchability while maintaining piezoelectric performance. A good example is polyvinylidene fluoride-trifluoroethylene/multiwall carbon nanotubes (PVDF-TrFE/MWCNT) [9]. For portable devices, some of the first reports considering this option were the fabrication of a Li-ion battery with a cathode of LiCoO_3_ and anode of TiO_2_, and piezoelectric polymer as the separator [10]. The mechanism of charging and energy conversion and storage is well explained from an electrochemical point of view. However, it is not discussed the problem of energy loss due to the non-regular events in the time sweep, i.e. the piezoelectric part generates an AC signal, but the battery part requires a DC signal. In the next stage of development, a few more papers report the integration of nanostructures with modifications of the involved materials, however, the moment with the proper power management remains unclear [11,12].

To integrate the supercapacitor functionality on a common substrate with the harvester element, the component could also be engineered to include conductive materials like graphene or carbon nanotubes in addition to the polymer composite. These materials can enable efficient charge storage and release capabilities within the flexible structure, as well as to enhance the element mechanical durability [13,14]. Another promising approach involves the utilization of MXenes, which offer high conductivity, large surface area, and excellent electrochemical properties [[15], [16], [17]]. However, the mechanical properties of MXenes can be a limiting factor in certain applications due to their unpredictable way of restacking the separate nanosheets [18]. Therefore, a careful selection of materials and design considerations is crucial to optimize the performance and durability of the integrated supercapacitor. Supercapacitor electrodes based on single crystal layered ZrX_2_ (X = S, Se) have been synthesized using chemical vapor transport method and exhibited stability, exceeding 90 % due to its greater conductive space observed from layer spacing [19]. In some cases, for biomedical sensors or drug delivery power supplies, bioimplantable materials are needed to realize the harvester and storage element [20,21]. The interface engineering between the electrode and the electrolyte is another important point of optimization. In particular, the electrolyte composition and its combination with the electrode are important for the efficient charge carriers transport and charge/discharge cycles. For this purpose, some research groups have focused on preparation of activated carbon and its energetic application for all-solid-state supercapacitors with PVA/KOH as a gel polymer electrolyte [22], or porous nanofibers membranes, exhibiting high ionic conductivity [23].

To ensure efficient charge storage and separation, a suitable dielectric material is crucial in supercapacitor design. Among various options, aluminum oxide (Al_2_O_3_) stands out as a reliable choice due to its easy controllable dielectric constant, resistance to electrical breakdown, and mechanical durability [24]. By effectively preventing short circuits and maintaining structural integrity, Al_2_O_3_ can be expected to contribute to the overall performance and reliability of the supercapacitor. Out of the electrochemical storage devices, aluminium oxide (Al_2_O_3_) is a durable material that can withstand high mechanical loading or harsh environments, making it potentially reliable choice for ensuring electrical isolation between the conductive layers in wearable supercapacitors. To enhance the performance of Al_2_O_3_-based supercapacitors, strategies can be employed to improve both the dielectric properties and the interfacial charge storage. Increasing the dielectric permittivity of Al_2_O_3_ can be achieved through techniques such as doping with suitable elements or creating specific microstructures [25]. Additionally, increasing the surface area and porosity of the Al_2_O_3_ can provide more active sites for charge carriers adsorption and desorption, leading to improved capacitance [26]. It is important to balance these approaches to optimize the overall performance of the supercapacitor, as increasing the dielectric permittivity might not necessarily lead to a significant increase in capacitance, and vice versa. To the best of our knowledge, the application of Al_2_O_3_ as a dielectric material in flexible supercapacitors has not been extensively explored. This study explores the novel integration of energy harvesters and supercapacitors to develop hybrid energy solutions on a flexible substrate for biosensors. We propose an approach to utilize Al_2_O_3_ as a high-performance dielectric material in flexible supercapacitor devices. The focus is on the investigation particularly the supercapacitor part and its behaviour according to the Al_2_O_3_ thickness. The motivation behind this research is to combine the energy-capturing capabilities of harvesters with the energy-storage efficiency of supercapacitors in a single structure, aiming to enhance overall energy utilization in wearable devices. The methodology involves designing and fabricating a prototype that integrates both technologies (harvesting and accumulating) to maximize energy capture and storage potential. The expected outcome of this research is to achieve a more robust hybrid energy system that can potentially advance the energy harvesting and storage low-power applications. Through this integrated approach, this study seeks to contribute towards compatibility of the technology for optimization of the sustainable energy solutions and promotion of the energy efficiency. After analysis of the market and studies of the separated components, it was found that the commercially available thin-film piezoelectric harvesters still use lead-zirconium titanate (PZT) with a relatively high thickness varying between 1 and 5 μm. Moreover, the supercapacitors still use pseudocapacitive charge storage mechanism, leading to voltage drop and higher equivalent series resistance (ESR). The used piezoelectric film in the proposed structure is eco-friendly piezoelectric polymer instead of lead-containing film and the thickness is reduced to ∼1 μm. The reduced quantity of active material providing piezoelectric power is compensated by doping of the polymer with multiwall carbon nanotubes (PVDF-TrFE:MWCNT), which create conductive network paths in the polymeric chain and enhance the electrical performance of this thinner film. The proposed design solved some of the existing challenges related to material compatibility, hindering the integration of the two component on a common substrate by now. The selected technological conditions ensure good adhesion between the piezoelectric polymer, conductive materials (like Ag and PEDOT:PSS doped with carbon nanotubes), and the dielectric layer (aluminium oxide). This order of the films ensures that voltage levels match and there are no significant resistive losses at the interfaces that worsens the effective energy harvesting and storage. Furthermore, while flexible materials are advantageous, they also need to exhibit sufficient mechanical stability and durability under repeated flexing and bending conditions. The mechanical properties difference of the films in the proposed structure is negligible, leading to reduced possibility for potential delamination, cracking, or functional degradation when subjected to stress. Regarding the supercapacitor part, aluminium oxide possesses sufficient dielectric permittivity allowing for a greater amount of charge to be stored within a given volume, and leading to higher capacitance values. It is produced with a high degree of porosity, providing a large surface area for charge storage. The thicker films of Al_2_O_3_ withstand a large number of voltage charge/discharge cycles without significant electrical degradation and doesn't store charge through redox reactions occurring on the surface of the electrode material. In addition, the mechanical stability corresponds to the needs of the wearables. All these aspects make the device superior to other similar investigated structures.

## 2 Materials and methods

### 2.1 Materials used for the piezoelectric harvesting and supercapacitor parts

Polyethylene terephthalate (PET) foil (Goodfellow, UK) with a thickness of 175 μm was cleaned in a 1:1 mixture of ethanol and water for 10 min and used as a substrate. Silver nanoparticles ink for printing (Sigma Aldrich, USA) was used for bottom electrode deposition. Polyvinylidene fluoride-trifluoroethylene (PVDF-TrFE) – based piezoelectric ink with piezoelectric coefficient d_33_ of ∼20 pC/N (Nanopaint, Portugal) was used as functional material for the energy harvester part. Poling conditions of 50 V/μm, 100 °C for 1 h, were set to enhance the piezoelectric properties. Multi-walled carbon nanotube (MWCNT, Sigma Aldrich, USA) with an average diameter of 65 nm was used as a particles source for obtaining a composite of PVDF-TrFE:MWCNT and current (respectively, power) gaining of the piezoelectric element. Poly(3,4-ethylenedioxythiophene)-poly(styrenesulfonate) (PEDOT:PSS, Merck, USA) ink with high conductivity grade was used for fabrication of intermediate (separating the harvester and storage part) and top electrodes. Dielectric layer, separating the electrodes of the supecapacitor and providing charges accumulation was produced from Al_2_O_3_ powder (SS Nano, USA) for thermal spraying. Graphene ink (Merck, USA) for enhancing the overall structure strength was inserted between the charge storage layer and the top carbon-based electrode.

### 2.2 Samples design and fabrication

**Fig. 1:**
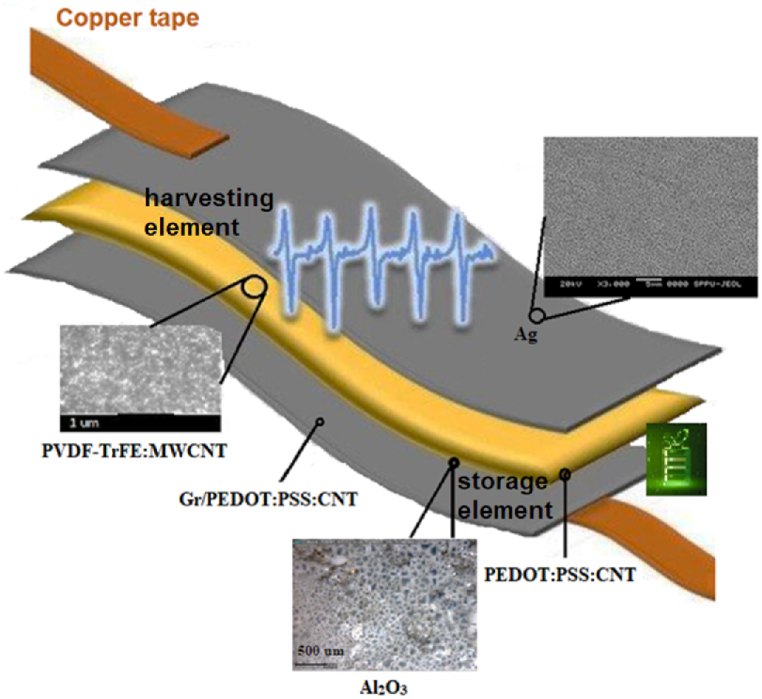
General view of the structure of the proposed flexible piezoelectric energy harvester and integrated supercapacitor.

Our proposed conceptual design involves a layer-by-layer fabrication process that incorporates a flexible substrate, conductive electrode layers, a piezoelectric polymer composite PVDF-TrFE:multiwall carbon nanotubes (MWCNT) for energy harvesting, and materials like graphene for supercapacitor functionality, electrically separated from the electrodes by aluminium oxide film. Utilizing advanced fabrication techniques such as ultrasonic spray deposition and pattern implementation, it was aimed at creating an energy system capable of capturing mechanical energy and storing it in reduced energy loss conditions. To integrate the supercapacitor functionality, the polymer composite is engineered to include conductive material like carbon nanotubes. These materials can enable efficient charge storage and release capabilities within the flexible structure. Low annealing temperature silver nanowires were grown as bottom electrode on the PET substrate. Piezoelectric composite PVDF-TrFE/MWCNT was spray coated on a photoresist grating pattern formed as a masking layer over the silver coating. Introducing such a pattern on the piezoelectric layer helps in the distribution of the mechanical stress more evenly across the layer, improving energy conversion and response. PEDOT:PSS:CNT was formed as the next layer in the stack for the intermediate electrode, forming the common terminal for the output of the harvester and the input of the storage element. This top electrode layer was patterned like interdigitated (IDT) by lift-off process (reverse photolithography) to enhance charge collection and distribution. Al_2_O_3_-based solution for rapid thermal annealing was used for the spray coating of the dielectric film onto the polymeric layer that serves as a seed. The growth on a non-crystalline base results in the formation of a highly porous, non-ordered dielectric coating, as is shown later in Fig. 4. Two different thicknesses of this film (400 nm and 750 nm) were produced. Thinner films than 400 nm were found lacking sufficient nucleation and growth of pores. This could be due to factors such as the limited amount of material. Without a porous structure, the effective surface area for charge storage is reduced, leading to a lower capacitance. Thicker films than 750 nm were found to dominate with their dielectric properties, hindering the charge transfer in the structure and isolating the piezoelectric element. The structures with intermediate thicknesses of aluminum oxide behave mostly like the thinner one. We studied the two extreme (boundary) cases of 400 nm and 750 nm that yielded significant and meaningful results. Graphene ink was sprayed on the Al_2_O_3_ as a cross-hatch pattern formed by photolithography, providing mechanical strength and stability while preventing direct contact between the conductive layers from the two sides of the dielectric layer and reducing the risk of short circuits. Finally, a top electrode layer made of a conductive material was added to complete the supercapacitor structure and enable external connections for charge/discharge. Because the deposition process has to be low-temperature, the PEDOT:PSS:CNT film was spray coated. A discrete power management system is connected as an intermediate unit, for the needs of initial testing and tuning of the energy conversion and storage process. It will be further made integral after establishing the proper mode of operation. Structure of the proposed design integrating energy harvesting and energy storage element is shown in Fig. 1.

Spray coating, also known as aerosol deposition, involves the atomization of a liquid precursor solution into fine droplets, which are then sprayed onto a substrate to form a thin film. Adjusting parameters such as spray rate, nozzle distance, and substrate temperature optimize the film uniformity and thickness for each layer. The spraying of the coatings was realized at a nozzle pressure of 3 bar and different temperatures, according to the volatility of the solvent (between ∼ 80 °C for the carbon-based composites and 100 °C for the separator dielectric). The area of the top collector electrode was 1 cm^2^, which makes the areal capacitance (F/cm^2^) to directly coincide with the measured capacitance.

### 2.3 Samples characterization

Methodology for piezoelectric voltage characterization and the setup for dynamic load of the samples are described elsewhere [27]. Piezoelectric response of the PVDF-TrFE:MWCNT-based harvester part of the element was recorded by a digital storage oscilloscope Tektronix TDS 1012B with activated noise rejection filter function. Supercapacitor voltage and its variations during charging/discharging cycles was recorded by using a multimeter Peakmeter PM8236 connected to a computer with Data Logger software. For the measurement of the retention capacitance Hioki Impedance analyser IM3590 was used. To guarantee the reliability of the measured results and proper characterization of the supercapacitor element, a reference signal from a signal generator was supplied first, before to activate the joint function of the two integrated elements. Stability of the capacitance and its charging voltage was tested by a lab-made electromechanical system for shock bending, where a motor is activated by a DC power source and thus regulates a vibrating cantilever. The samples were attached on the top of this cantilever and loaded with a mass-force equivalent to 2 kg, which is typical maximal loading expected in real-time operational conditions if assumed wears and bends of the elbows or knees. All the data are averaged from three identical samples of each type, having two electrode top segments (i.e. six samples in total for each thickness). For impedance matching between the piezoelectric converter and the supercapacitor, an original power management system was developed, which is described below.

## 3 Results and discussion

### 3.1 Power management circuit configuration

**Fig. 2:**
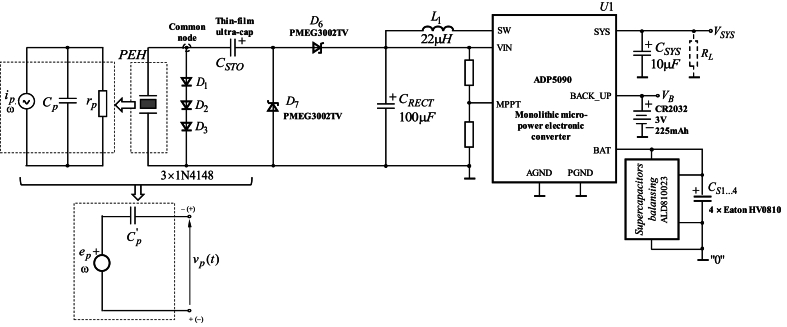
Circuit diagram of the power management system integrated with the simplified equivalent circuit of the piezoelectric energy harvester and thin-film low-power supercapacitors.

Fig. 2 shows the synthesized circuit diagram of the power management system designed for converting and boosting input AC voltage. Furthermore, the input AC voltage is generated by integrating the thin-film piezoelectric energy harvesters and thin-film supercapacitors (the two elements are configured in series and have a common node that is externally accessible), presented in the above sections. In this case, the thin-film supercapacitors are charged by an AC current with a non-sinusoidal waveform, since the piezoelectric elements vibrate at frequencies below their resonant frequency. As a result, the voltage across the supercapacitors increases approximately monotonically, and its limitation is achieved by a voltage limiter. It is composed of three series-connected Si diodes of type 1N4148. The maximum voltage for this configuration is given by *V*_*limit*_ = 3 × *V*_*d*_ ≈ 1.8*V*, where *V*_*d*_ ≈ 0.6*V* is the voltage drop across an individual Si diode. To obtain a DC output voltage, the thin-film supercapacitor is integrated as a part of an AC-DC converter that includes a voltage-doubler rectifier and a low-power DC-DC converter. The voltage limiting mechanism, using series-connected diodes, provides possibility of subsequent operation with some of the low-power DC-DC converters, enabling compatibility. The typical maximum input voltage for these converters, does not exceed 3.6*V*.

The equivalent circuit of the system of a piezoelectric generator and a thin-film supercapacitor can be represented, according to Thevenen's theorem, as a voltage source and an equivalent capacitor $C_{p}^{\prime }\approx C_{P}+C_{STO}$ connected in series. Then, for the maximum value of the open-circuit voltage *v*_*p*_(*t*) (assuming that the internal resistance *r*_*p*_ of the generator is negligible) can be expressed as

(1) $$V_{pm,0}^{\prime }=\frac{I_{pm,0}^{\prime }}{\omega C_{p}^{\prime }}\text{,}$$

where $I_{pm,0}^{\prime }$ denotes the maximum value of the input current through the equivalent capacitor with a capacitance $C_{p}^{\prime }$ and ω is the frequency of the voltage fluctuations, according to the mechanical deflection.

#### 3.1.1 Voltage-doubler rectifier

The electronic circuit of a passive AC-DC converter is used to limit the voltage fluctuations. The passive AC-DC converter operates as a voltage rectifier, characterized by a minimal voltage drop across the diodes. It prevents reverse current flow when the voltage *V*_*LIMIT*_ is lower than the voltage *V*_*RESTIFIED*_ [28,29]. Given that the amplitude of the voltage may be insufficient to be directly applied to the DC-DC converter, a voltage rectifier with a multiplication factor of two, commonly referred to as a voltage doubler circuit, is employed [30,31]. In this case, it is necessary to use diodes with a low voltage drop when operate in forward direction. For a single sample, the voltage is lower than 300 … 350 mV, which requires the use of diodes with a forward direction voltage of 100 … 120 mV at a current of approximately 0.1 mA. The voltage multiplier circuit, or voltage-doubler rectifier, incorporates two Schottky diodes (*D*_6_ and *D*_7_) type PMEG3002TV along with two capacitors (*C*_*P*_ and *C*_*RECT*_). The selected Schottky diodes exhibit a low voltage drop in the forward mode of operation (≈130 mV at a forward current of 0.1 mA) and a low reverse current (≈3 μ*A* at a reverse voltage of up to 10*V*).

The operational principle of the voltage multiplier can be described as follows: during the *first half-cycle* for which the polarity of the equivalent voltage source is shown without brackets, the capacitor $C_{p}^{\prime }$ is charged through the $D_{6}$ to the amplitude of the input voltage – $V_{C1}=V_{pm,0}$. Under this polarity, the diode $D_{7}$ is in forward operation mode. In the subsequent *half-cycle* when the voltage reverses its polarity (signs in brackets), the diode $D_{7}$ is turned off. The sum of the capacitor voltage and the amplitude of the input voltage is applied to this diode. In this half-cycle, the diode $D_{6}$ is turned on and the capacitor $C_{RECT}$ is charged alongside the capacitor $C_{p}^{\prime }$, transferring a portion of its electrical energy until reaching a voltage, equal to the sum of the input voltage and the voltage of $C_{p}^{\prime }$. As a result, the output voltage of the rectifier can be expressed as $V_{C_{RECT}}=V_{pm,0}^{\prime }+V_{C_{P}^{\prime }}\approx 2V_{pm,0}^{\prime }=600...700mV$. (2)

The maximum value of the average electrical power that is extracted from the piezoelectric element occurs when the multiplier voltage *v*_*p*_(*t*) is one-half of the peak no-load voltage. It can be determined, according to

(3) $$P_{av,\max}=\frac{C_{p}^{\prime }\omega V_{C_{RECT}}^{2}}{2\pi }\approx 27mW\text{,}$$

where ω=2π×50Hz≈314rad/s and $C_{p}^{\prime }\approx 1.5mF$.

In the voltage doubler circuit, both diodes operate at the same maximum reverse voltage that is twice the amplitude of the input voltage. The two capacitors are charged to different voltage levels. Upon connecting a load to the output of the circuit, the charge level of the capacitors decreases, resulting in a corresponding decrease in the output voltage. This can be attributed to the internal resistance *r*_*p*_ of both the voltage source and the diodes, as well as the principle of operation of the circuit. In this case, each capacitor derives part of its charge energy from the previous capacitor, which is partially discharged, and its voltage is reduced. When using the *C*_*STO*_, a higher voltage is delivered, thereby facilitating an increase in the output current.

#### 3.1.2 Micro-power DC-DC conversion circuit

An electronic system of a micro-power DC-DC converter is chosen for the stabilization of the rectified voltage. It ensured a constant value of the output voltage, when the input voltage changed, or when the load at the output port changed. The analysis of scientific publications for the last five years in the field of low-power energy harvesting systems showed a great variety of electronic circuits. Moreover, some of them are implemented as monolithic integrated circuits [[32], [33], [34], [35], [36]], and others are realized with discrete components [[37], [38], [39], [40], [41], [42], [43]]. The majority of the reported circuit configurations have a relatively high value of energy efficiency (>60 %), with an output power in some circuits, achieving several mW, and a figure of merit (FoM) of up to 400 … 500 %. In all proposed circuits for the used DC-DC converters, the input voltage must be greater than 1.2 … 1.5V, and they must be self-powered. For the proposed sources of electrical energy in this work, the input voltage of the DC-DC converter that was achieved is as low as 1V. Furthermore, for the proposed electronic system, it is necessary to maintain the continuity of the device's operation. This can be achieved by including an additional backup battery in the structure of the power management circuit. The additional backup battery has to provide electrical energy when the energy of the piezoelectric generator is not sufficient. For the implementation of a micro-power DC-DC conversion circuit, a monolithic integrated circuit ADP5090 [44] was chosen.

The ADP5090 is equipped with a low-power DC-to-DC step-up (or boost) converter designed to operate within an input voltage range from 80 mV up to 3.6*V*, after the cold start is completed. The minimum input voltage for a cold start condition (at *V*_*SYS*_ = 0*V*) has a typical value of 380 mV. The current consumption in quiescent mode of operation is less than 400 nA. Furthermore, analysis of the datasheet for the IC ADP5090 indicates that the coefficient of the electrical energy efficiency can exceed 80 % when the input voltage exceeds 600 mV and the input current is around 100 μ*A*. These operational characteristics are particularly relevant for the proposed system's specific application because the thin-film piezoelectric generators typically operate with a maximum input voltage in the same range. Additionally, in wearable electronics, there may be intervals of low physical activity, when the input voltage can fall below 100–200 mV, however, the selected IC ADP5090 remains operational under these conditions.

The evaluation board for the IC ADP5090 – EVAL-ADP5090 [45] is used to carry out preliminary studies in the implementation of an energy harvesting system. For the evaluation board EVAL-ADP5090, the output voltage is fixed and has the following value

(4) $$V_{SYS}=\frac{3}{2}V_{REF}\left(1+\frac{R_{3}}{R_{6}}\right)\approx \frac{3}{2}\times 1.2V\times \left(1+\frac{4.87M\Omega }{5.11M\Omega }\right)\approx 3.5V\text{,}$$

where $V_{REF}$ is the reference voltage typically equal to 1.2V, $R_{3}=4.87M\Omega$ and $R_{6}=5.11M\Omega$.

To ensure the value of the output voltage, four low-power supercapacitors type Eaton HV0810 (each with a nominal capacity of 1*F* and maximum voltage across the two terminals of 2.7*V*) with a voltage balancing system ALD810023 (from Advanced Linear Devices), are connected to the BAT terminal of the ADP5090. The energy efficiency of the ADP5090 can attain 90 % at an input voltage at least 1.4*V*. At input voltage around 0.6*V*, the efficiency is above 80 %. In addition to the supercapacitors at a BACK_UP terminal, a backup battery type CR2032 with a voltage value of 3V is connected. This backup battery facilitates the cold start process when the piezoelectric generator is initially turned on and VSYS = 0 or to ensure the electrical power delivered to the load, connected to the SYS terminal, during periods of mechanical vibrations attenuation, resulting in low generated input current. Therefore, on the one hand, continuous operation can be maintained. As a result, the output current of the electronic system can exceed 100 mA (determined by the capacity of the backup battery). On the other hand, incorporating charging circuits for energy harvesting from the environment into the design of micro-power sources for wearable electronic devices can significantly extend the lifespan of the primary battery, thereby prolonging the intervals between battery replacement events.

The capacitance of the developed thin-film supercapacitors was measured using an impedance analyzer Hioki IM3590. For this purpose, a sinusoidal signal with a frequency of 10Hz and an amplitude of 1*V* is supplied from the internal AC voltage generator. Since the impedance analyzer allows a 4-terminal pair configuration, the voltage is measured between H_POT_ and L_POT_ terminals using a built-in AC voltmeter, and the current is measured between H_CUR_ and L_CUR_ terminals flowing through the tested element.

**Fig. 3:**
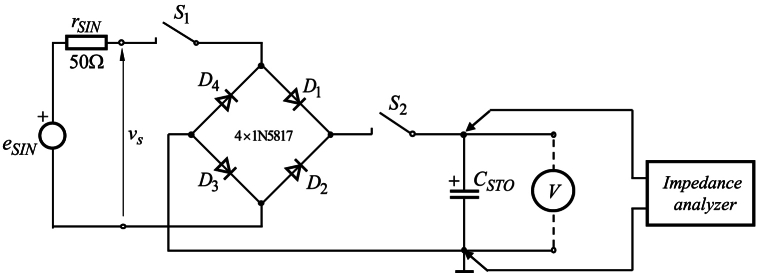
Electronic circuit for measurement of the capacity of the developed thin-film supercapacitors by impedance analyser.

The electronic circuit for testing the capacity of the developed thin-film supercapacitors is shown in Fig. 3. The measurement procedure was conducted in two steps so that the capacity of the supercapacitor can be determined for a certain number of charge and discharge cycles. During the time interval of the first step, switch *S*_1_ was closed, while switch *S*_2_ remained open, allowing the supercapacitor charging to a voltage of $V_{STO,0}=V_{pm,0}^{\prime }-2V_{D}$, where *V*_*D*_ is the voltage drop of the rectifying diode. The input signal was generated using function generator MPF3060, and the voltage across the supercapacitor was measured by digital multimeter PM8236. Then, during the time interval of the second step, the switch *S*_1_ was open, while *S*_2_ was closed. An AC voltage was then applied from the analyzer to measure the capacitor with the residual charge on it. This methodology enables the evaluation of the operational performance of the engineered supercapacitor during multiple charging and discharging cycles without significant alteration in its capacitance.

**Fig. 4:**
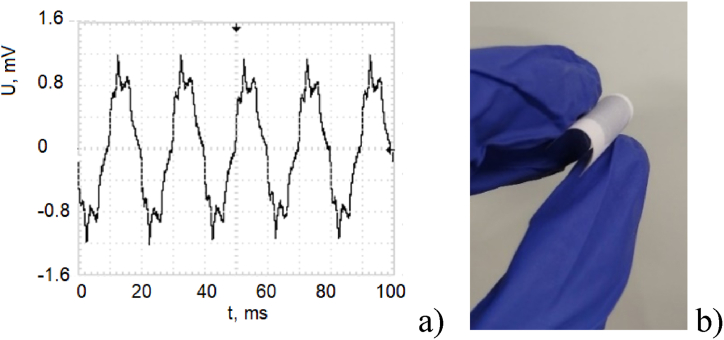
Piezoelectric harvesting part: a) voltage output of the piezoelectric generator under a 50 g/cm^2^ applied load; b) photograph of one of the fabricated piezoelectric generator devices, after integration with the supercapacitor part.

### 3.2 Device characterization

The piezoelectric element exhibited optimal performance within a mass loading range of 10–110 g/cm^2^, enduring over 5000 bending cycles without degradation. This loading range aligns with typical human motion, generating forces when attached to limbs. Under these conditions, the element produced voltage outputs between 1 and 2 V, and current between 800 μA and 1.2 mA, depending on the excitation frequency (10–50 Hz). This sensitivity to external vibrations, coupled with human motion, makes the device suitable for various applications working with external vibrations. The produced electrical power varies between 800 μW and 2.4 mW, which fall in the range of suitable values to supply low-power, wearable biosensors, and can be further increased with the mass loading increase. Fig. 4a illustrates the sinusoidal waveform generated under moderate loading conditions, while Fig. 4b shows a photograph of a fabricated device. While the piezoelectric component has been extensively studied in previous work [46], this research focuses on optimizing the supercapacitor element, particularly the impact of dielectric film thickness.

**Fig. 5:**
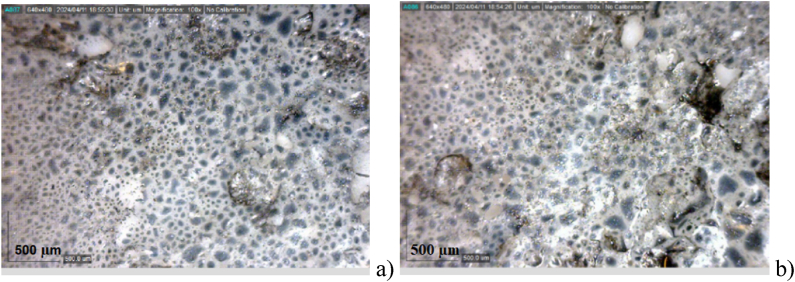
Optical microscopic images with magnification ×100, revealing the interconnected pore network of sprayed Al_2_O_3_ films with an average thickness of: a) 400 nm; b) 750 nm.

Fig. 5a and b presents microscopic images of Al_2_O_3_ films with thicknesses of 400 nm and 750 nm, respectively. The underlying polymer film serves as a seed for the growth of a non-ordered (in this case porous) Al_2_O_3_ structure, as reported in Ref. [47]. Image analysis using ImageJ revealed that the thicker film exhibits a higher degree of porosity (10 %) compared to the thinner film (4 %) for the snapped area (10 μm ⅹ 10 μm**)**. The defects are considered size or shape deviations of more than 5 % from the average or irregular distribution of the pores. This increased porosity, making its surface-to-volume ratio greater, arising from the growth of the thicker film on an already porous substrate, provides a larger surface area for charge accumulation and storage. Consequently, the 750 nm Al_2_O_3_-based supercapacitor is expected to exhibit superior performance.

The charging behavior of a supercapacitor is crucial for understanding its performance and optimizing its design. By analyzing the voltage-time profiles during the charging process, valuable insights can be gained into the device's charging dynamics and efficiency.

**Fig. 6:**
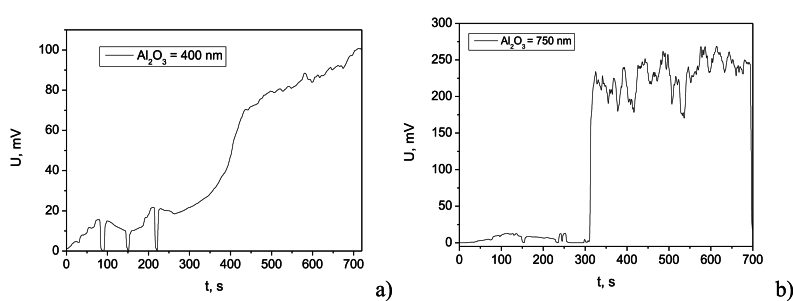
Supercapacitor voltage as a function of time during the charging process of samples with two different thicknesses of the Al_2_O_3_ dielectric layers: a) charging profile at Al_2_O_3_ 400 nm; b) charging profile at Al_2_O_3_ 750 nm.

Fig. 6a and b illustrate the charging curves for supercapacitors with Al_2_O_3_ dielectric thicknesses of 400 nm and 750 nm, respectively, at an input voltage of 2 V and a frequency of 10 Hz. The slope of the voltage-time curve provides insights into how quickly the supercapacitor is charging. The steeper slope of the charging curve for the 750 nm device indicates a faster charging rate compared to the 400 nm device, showing gradual slope and suggesting slower charging. The 750 nm device achieved a charging time of 6 s, while the 400 nm device required 310 s. The transient process at the thinner dielectric process is longer, as can be seen from the voltage drops until a stable value is established. The higher voltage achieved by the 750 nm device (average of 200 mV) compared to the 400 nm device (average of 80 mV) suggests a higher capacitance and lower internal resistance. This is consistent with the observed faster charging rate and the higher porosity of the thicker dielectric film.

**Fig. 7:**
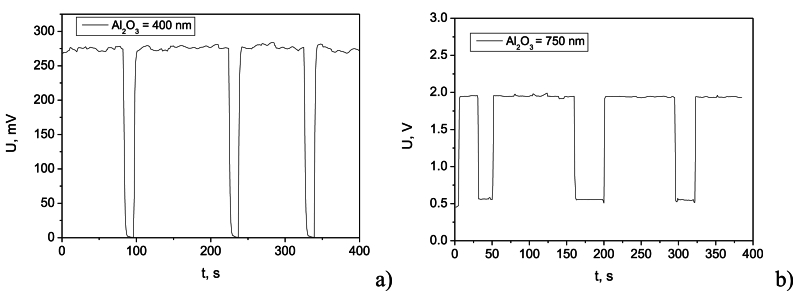
Variation of the supercapacitor output voltage at charging-discharging cycles for samples with Al_2_O_3_ dielectric film having thickness of: a) 400 nm; b) 750 nm.

The voltage-time curves during charging-discharging cycles, depicted in Fig. 7a and b, reveal variations in the charging process, manifested as ripples in the voltage profile. These ripples can be attributed to factors such as contact resistance and capacitance variations. The 750 nm Al_2_O_3_-based supercapacitor exhibited an average charging voltage of 1.94 V with a voltage ripple of ±0.59 mV. The 400 nm device achieved an average charging voltage of 275 mV with a voltage ripple of ±0.50 mV. The thinner storage element exhibited a full discharge to nearly 0 V as a low load resistance, while the thicker element reached a minimum voltage of 0.5 V. Understanding the saturation point of the supercapacitor is crucial to prevent overcharging and device degradation. Observing a plateau in the voltage-time curve indicates that the supercapacitor is nearing full charge. The 400 nm device reached its saturation point at approximately 274 mV after the initial transient period, while the 750 nm device reached saturation at approximately 1.94 V.

**Fig. 8:**
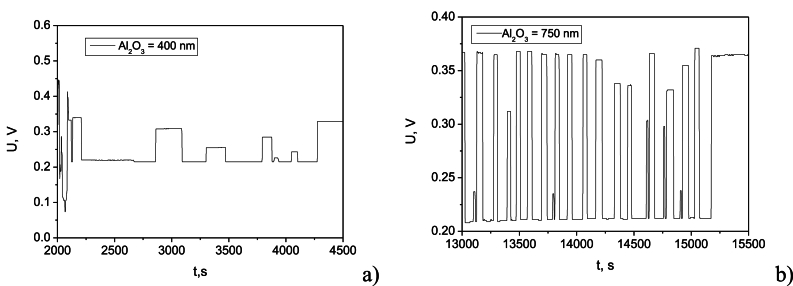
Number of charge/discharge cycles of the supercapacitor before electrical failure: a) at Al_2_O_3_ of 400 nm; b) at Al_2_O_3_ of 750 nm.

The long-term charge/discharge cycling stability of a supercapacitor is a critical performance metric for determination of its life cycle. Examining the voltage fluctuations during cycling can reveal the supercapacitor's ability to maintain a stable voltage output over repeated charge/discharge cycles. Voltage stability is essential for ensuring consistent performance in applications requiring steady power delivery. Fig. 8a and b illustrate the cycling stability of the devices with 400 nm and 750 nm Al_2_O_3_ dielectric thicknesses, respectively. Examining the voltage fluctuations during cycling can reveal the supercapacitor's ability to maintain a stable voltage output over repeated charge/discharge cycles. Voltage stability is essential for ensuring consistent performance in applications requiring steady power delivery. As is seen in Fig. 8a, the storage device with the thinner dielectric deviates from the regular cyclic performance after 2000 cycles and can resist up to 4500 cycles, after which stopped change after switching on and off. The structure with the thicker dielectric film showed more stable performance and started to deviate after 13000 cycles, resisting up to 15500 before stopping to respond to the switching input voltage (Fig. 8b). The test was conducted at a reduced input voltage to avoid degradation due to overvoltage and to differentiate the response to the switching cycles from the maximum allowable voltage limit, which additionally may exhaust the structure. Overall, this means that the thicker dielectric film leads to a more durable and reliable storage device that can handle more cycles of charging and discharging before failing compared to the thinner dielectric. When the layer is involved in charge storage and transfer, dielectric losses may occur, especially if the frequency of the applied signals is high. With repeated charge and discharge cycles, this can lead to additional energy losses in the form of heat. Instability due to heat fluctuations is low probable, because of the thermal stability of aluminum oxide. In addition, over time, the PEDOT:PSS:CNT layer can develop leakage currents, especially in humid or high-temperature environments. These currents can drain energy from the supercapacitor, thus lowering the overall efficiency of the energy storage system. The combination of the high dielectric permittivity and controlled porosity can contribute to uniform interfacial resistance between the Al_2_O_3_ layer and the PEDOT:PSS:CNT layer. By maintaining a better-defined interface and preventing excessive charge accumulation at the dielectric-electrode boundary, the porous Al_2_O_3_ can help in suppressing leakage currents caused by localized charge build-up.

**Fig. 9:**
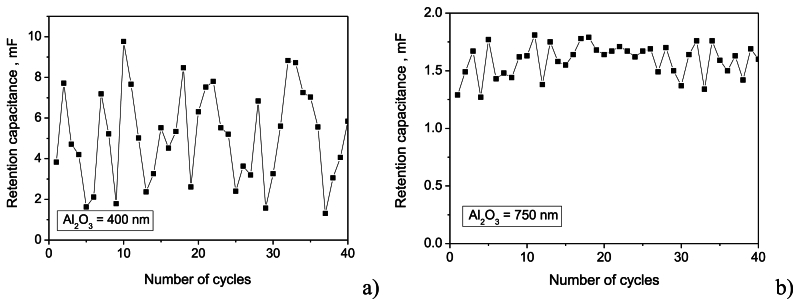
Retention capacitance as a function of the charge-discharge cycles number for supercapacitors with two different thicknesses of the Al_2_O_3_ dielectric film: a) 400 nm; b) 750 nm.

Retention capacitance quantifies a device's ability to retain charge over time. For capacitive storage devices, a higher retention capacitance indicates how effectively the system can store a charge without significant leakage or discharge. As depicted in Fig. 9a and b, the device with the thicker Al_2_O_3_ dielectric exhibited superior retention capacitance, maintaining a stable value of 1.63 mF with a standard deviation of 0.14 mF over multiple charge-discharge cycles. In contrast, the thinner Al_2_O_3_ device, while initially exhibiting a higher average retention capacitance of 5.21 mF, displayed significant variability with a standard deviation of 2.29 mF. This suggests that the thicker dielectric layer provides better charge trapping and reduced leakage currents, resulting in improved retention performance. Considering the average retention capacitance and the generated voltage, the device with the 750 nm Al_2_O_3_ dielectric demonstrated an estimated energy storage density of 3 mJ/cm^2^. This value highlights the potential of this device for energy harvesting and storage applications, because it is in line and even greater than reported in the literature relative to the area [48]. The strong variation around the average value for the thinner dielectric film makes this structure inappropriate for a reliable energy storage process.

**Fig. 10:**
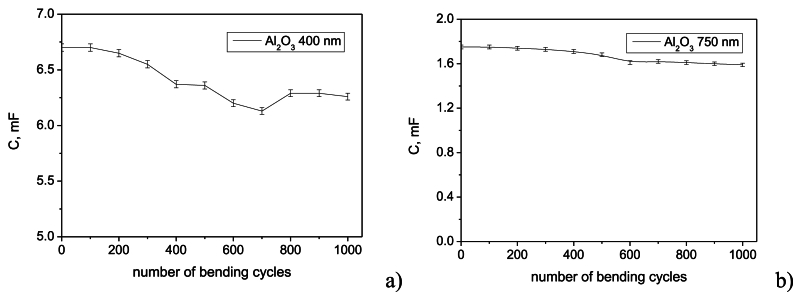
Capacitance stability of the supercapacitors under compressive-tensile (bending) repeating loading cycles for device with different Al_2_O_3_ thickness: a) 400 nm; b) 750 nm.

To assess the mechanical stability of the device, cyclic bending tests were performed at a frequency of less than 20 Hz, simulating the repetitive stress experienced during typical human motion because of the limited range of motions at these joints. The device was subjected to a load of 2 kg, representing a realistic load for a wearable device attached to the elbow or knee. Fig. 10a and b illustrate the device's stability under repeating compressive/tensile loading for the devices using two Al_2_O_3_ thicknesses, respectively. The device demonstrated excellent mechanical robustness, withstanding numerous cycles of deformation without significant degradation.

The 400 nm Al_2_O_3_ device exhibited a more stable performance, with a modest 7 % decrease in capacitance after 1000 bending cycles. The 750 nm device experienced ∼9 % decrease in capacitance over the same number of cycles. The increased thickness of the dielectric layer in the 750 nm device, while initially providing higher capacitance, is slightly more susceptible to mechanical stress. However, it doesn't lead to a significant decrease in performance over time and it is comparable with the values reported in the literature [49]. The reason for retain their energy storing capacity so high (93 % and 91 %) after multiple bending can be ascribed to the reinforcing contribution of graphene, which prevent delamination between the supplying harvesting element.

## 4 Conclusions

The power management device between the harvesting and storage element in case of alternative electrical signal generation is crucial for minimizing energy losses. In this work, a specific combination of materials along with the methods used to integrate them into a flexible, hybrid system for energy harvesting and storage is unique. The application of aluminum oxide (Al_2_O_3_) as an insulating layer between conductive electrodes is a novel choice. Al_2_O_3_ offers good mechanical strength and high dielectric constant, which improve the stability and performance of supercapacitors in flexible devices. Employing fabrication methods such as ultrasonic spray deposition and pattern implementation to create the multilayered structure also distinguishes the novel approach. Such techniques result in a more uniform distribution of materials and better interface engineering, reducing the energy losses during energy conversion and energy transferring. Utilizing materials like Al_2_O_3_ and Graphene in the proposed configuration provides layers that can enhance the durability and stability of the components under mechanical stress or thermal fluctuations, especially if the frequency of the applied signals is high (meaning frequent vibrations or other mass-loading), extending the lifetime and reliability of the entire system. PVDF-TrFE:MWCNT composite configuration enhances piezoelectric properties facilitating better charge transport and introduces conductivity via MWCNTs, offering also improved flexibility. PEDOT:PSS with CNT layer acts as both a charge collector and a buffer that balances the differences in mechanical and electrical properties between the piezoelectric and dielectric layers. It aids in stabilizing voltage levels during energy transfer, because of the reduction of interface resistance. Doping levels or composite ratios of the PEDOT:PSS:CNT layer can be adjusted to optimize the capacitance of the overall element to suit specific application needs. The inclusion of this layer as a conductive intermediate pin allows for better management of transient loads. If the system experiences sudden changes in load demand, the internal pin can facilitate faster modulation of the charge supply, enhancing system responsiveness. The proposed designs do not consider intermediate electrical pin in their configurations. Graphene is not commonly used in conventional piezoelectric-supercapacitor setups, which may utilize carbon blacks. It is used here to increase the conductivity and surface area for charge storage within the supercapacitor structure. A two-stage interface circuit combining the use of discrete components with commercially available specialized integrated circuits for AC to DC voltage conversion and transmission has been developed. Moreover, the first (input) stage provides AC to DC voltage conversion by approximately doubling the rectified voltage using an amplitude-limiting voltage-doubler rectifier implemented using micro-power Schottky diodes with a minimum value of the reverse current. The second (output) stage is a micro-power DC-to-DC converter, stabilizing the rectified voltage and controlling the charging process of low-voltage energy storage elements through a charge-balancing system. The developed interface circuit built in this way contains few passive discrete components, minimal physical sizes of the electronic device, and guarantees to obtain an optimal value of the energy efficiency coefficient. The proposed AC to DC boosting converter provide voltage with an appropriate value to the supercapacitor, although the irregular signal comes from the piezoelectric element. The spray coating technique provides excellent compatibility of the layers involved without damage or partial dissolution of the sublayers, which was demonstrated by the testing of the two elements separately and measurement of the main characteristics, typically describing their behaviour. The growth of a thicker dielectric film leads to a more stable performance of the supercapacitor part. Thinner films have a lower surface area available for charge storage, limiting the amount of stable charge it can hold. Thinner films probably result in higher internal resistance within the supercapacitor, which can be a reason for the longer transient process and larger voltage ripples during charge/discharge cycles. Thinner films may have reduced dielectric strength, making them more susceptible to breakdown under high voltages or prolonged cycling, which could impact the stability and susceptibility to degradation over repeated charge/discharge cycles, leading to a decline in capacitance retention and overall performance consistency over time.

Overall, the development and exploration of integrated energy harvesting and storage systems present an opportunity to advance the field of flexible electronics, sustainable energy solutions, and multi-functional energy devices. The interdisciplinary nature of such research, combining materials science, energy conversion, and device engineering, could pave the way for future innovations and applications in the realm of self-sufficient and efficient energy technologies.

Future work will be directed to the optimization of the technology to achieve more stable results and toovercome the current limitation of the proposed element – the unipolar power supply due to the lack of positive ions source. The potential difference is formed like the negative charge as compared to ground, which may be limitation for some electronic circuits requesting bipolar power supply. Also, the electronic circuit for power management will be realized onto a polyimide copper coated foil for fabrication of a flexible printed circuit board for the complete integral device, free of discrete components.

## CRediT authorship contribution statement

**Mariya Aleksandrova:** Writing – original draft, Visualization, Methodology, Investigation, Formal analysis, Data curation, Conceptualization. **Ivaylo Pandiev:** Writing – review & editing, Methodology, Investigation, Funding acquisition.

## Data availability

Data will be made available on request.

## Ethics statement

The work described has not been published previously. The article is not under consideration for publication elsewhere. The article's publication is approved by all authors and by the responsible authorities where the work was carried out. If accepted, the article will not be published elsewhere in the same form, in English or in any other language, including electronically without the written consent of the copyright-holder.

## Funding sources

This work has been accomplished with financial support by the European Regional Development Fund within the Operational Programme “Bulgarian National Recovery and Resilience Plan”, procedure for direct provision of grants ”Establishing of a network of research higher education institutions in Bulgaria”, and under Project BG-RRP-2.004-0005 “Improving the research capacity anD quality to achieve international recognition and resilience of TU-Sofia (IDEAS)”

## Declaration of competing interest

The authors declare that they have no known competing financial interests or personal relationships that could have appeared to influence the work reported in this paper.

## References

[1] Fagiolari Lucia, Sampò Matteo, Lamberti Andrea, Amici Julia, Francia Carlotta, Bodoardo Silvia, Bella Federico (2022). Integrated energy conversion and storage devices: interfacing solar cells, batteries and supercapacitors. Energy Storage Mater..

[2] Zhong Y., Xia X., Mai W., Tu J., Fan H.J. (2017). Integration of energy harvesting and electrochemical storage devices. Advanced Materials Technologies.

[3] Pu X., Hu W., Wang Z.L. (2018 Jan). Toward wearable self-charging power systems: the integration of energy-harvesting and storage devices. Small.

[4] Huang S., Gao Y., Hu Y., Shen F., Jin Z., Cho Y. (2023 Oct 2). Recent development of piezoelectric biosensors for physiological signal detection and machine learning assisted cardiovascular disease diagnosis. RSC Adv..

[5] Arshad H., Raza W., Mehmood A., Jalees S., Ao L., Deng Y., Ramie A., Cai X., Liu D. (2024). Ionic potency regulation of coagulation bath induced by saline solution to control over the pore structure of PBI membrane for high-performance lithium metal batteries. J. Energy Chem..

[6] Hussain A., Luo Y., Li T., Zhang H., Mirza S., Zhang H., Li X. (2020). Stop four gaps with one bush: versatile hierarchical polybenzimidazole nanoporous membrane for highly durable Li–S battery. ACS Appl. Mater. & Interf.

[7] Ali Faizan, Raza Waseem, Li Xilin, Gul Hajera, Kim Ki-Hyun (2019). Piezoelectric energy harvesters for biomedical applications. Nano Energy.

[8] Kim J., Campbell A.S., de Ávila B.E.F. (2019). Wearable biosensors for healthcare monitoring. Nat. Biotechnol..

[9] Aleksandrova M., Tsanev T., Kadikoff B., Alexandrov D., Nedelchev K., Kralov I. (2023). Piezoelectric elements with PVDF–TrFE/MWCNT-aligned composite nanowires for energy harvesting applications. Crystals.

[10] Xue X., Wang S., Guo W., Zhang Y., Wang Z.L. (2012). Hybridizing energy conversion and storage in a mechanical-to-electrochemical process for self-charging power cell. Nano Lett..

[11] Xue X., Deng P., He B., Nie Y., Xing L., Zhang Y., Wang Z.L. (2013). Flexible self-charging power cell for one-step energy conversion and storage. Adv. Energy Mater..

[12] Ramadoss A., Saravanakumar B., Lee S.W., Kim Y.-S., Kim S.J., Wang Z.L. (2015). Piezoelectric-driven self-charging supercapacitor power cell. ACS Nano.

[13] Fekri Aval L., Ghoranneviss M., Behzadi Pour G. (2018). High-performance supercapacitors based on the carbon nanotubes, graphene and graphite nanoparticles electrodes. Heliyon.

[14] Sheng H., Ma Y., Zhang H., Yuan J., Li F., Li W., Xie E., Lan W. (2024). Integration of supercapacitors with sensors and energy-harvesting devices: a review. Adv. Mater. Technol..

[15] Fu Z., Wang N., Legut D., Si C., Zhang Q., Du S., Germann T.C., Francisco J.S., Zhang R. (2019). Rational design of flexible two-dimensional MXenes with multiple functionalities. Chem. Rev..

[16] Anasori B., Lukatskaya M.R., Gogotsi Y. (2017). 2D metal carbides and nitrides (MXenes) for energy storage. Nat. Rev. Mater..

[17] Kumar K.S., Choudhary N., Jung Y., Thomas J. (2018). Recent advances in two-dimensional nanomaterials for supercapacitor electrode applications. ACS Energy Lett..

[18] Ibrahim Y., Mohamed A., Abdelgawad A.M., Eid K., Abdullah A.M., Elzatahry A. (2020). The recent advances in the mechanical properties of self-standing two-dimensional MXene-based nanostructures: deep insights into the supercapacitor. Nanomaterials.

[19] Habib M., Ullah S., Khan F., Rafiq M.I., Balobaid S.A., Alshahrani T., Muhammad Z. (2023). Supercapacitor electrodes based on single crystal layered ZrX_2_ (X = S, Se) using chemical vapor transport method. Mater. Sci. Eng. B: Solid-State Mater. Adv. Technol..

[20] Sheng H., Zhang X., Liang J., Shao M., Xie E., Yu C., Lan W. (2021). Recent advances of energy solutions for implantable bioelectronics. Adv. Healthcare Mater..

[21] Sheng H. (2023). A soft implantable energy supply system that integrates wireless charging and biodegradable Zn-ion hybrid supercapacitors. Sci. Adv..

[22] Ahmad A., Gondal M.A., Hassan M., Iqbal R., Ullah S., Alzahrani A.S., Memon W.A., Mabood F., Melhi S. (2023). ACS Omega.

[23] Hussain A., Mohamed M.M., Aijaz M.O., Karim M.R., Aziz M.A. (2024). Electrospun PEI/PAN membrane for advanced Zn ion hybrid supercapacitors. J. Energy Storage.

[24] Ponticorvo Eleonora, Galvagno Sergio, Portofino Sabrina, Borriello Carmela, Tammaro Loredana, Iovane Pierpaolo, Rametta Gabriella, Sarn Maria (2021). Alumina based electrode for stable and improved supercapacitor applications. CHEMICAL ENGINEERING TRANSACTIONS.

[25] Wu Lin-Jung, Wu Jenn-Ming (September 2007). Improved dielectric properties of -doped thin films for tunable microwave applications. Appl. Phys. Lett..

[26] Gholizadeh Z., Aliannezhadi M., Ghominejad M. (2023). High specific surface area γ-Al2O3 nanoparticles synthesized by facile and low-cost co-precipitation method. Sci. Rep..

[27] Aleksandrova M., Kolev G., Vucheva Y., Pathan H., Denishev Kr (2017). Characterization of piezoelectric microgenerator with nanobranched ZnO grown on a polymer coated flexible substrate. Appl. Sci..

[28] Dallago E. (2008). *Proc. Of IEEE International Symposium on Circuits And Systems* (ISCAS).

[29] Aktakka E.E., Najafi K. (2014). A micro inertial energy harvesting platform with self-supplied power management circuit for autonomous wireless sensor nodes. IEEE J. Solid State Circ..

[30] Tietze V., Schenk Ch (2008). Electronic Circuits.

[31] Stefanov N. (2002). Power Supply Devices. Tehnika: Sofia, Bulgaria.

[32] Li S., Roy A., Calhoun B.H. (2019). In Proc. Of the 2019 Symposium on VLSI Circuits.

[33] Du S., Jia Y., Zhao C., Amaratunga G.A.J., Seshia A.A. (2019). A fully integrated split-electrode SSHC rectifier for piezoelectric energy harvesting. IEEE J. Solid State Circ..

[34] Chamanian S., Muhtaroğlu A., Külah H. (2020). A self-adapting synchronized-switch interface circuit for piezoelectric energy harvesters. IEEE Trans. Power Electron..

[35] Çiftci B., Chamanian S., Koyuncuoğlu A., Muhtaroğlu A., Külah H. (2020). A low-profile autonomous interface circuit for piezoelectric micro-power generators. IEEE Transactions on Circuits and Systems I: Regular Papers.

[36] Chamanian S., Çiftci B., Muhtaroğlu A., Külah H. (2021). A self-powered and area efficient SSHI rectifier for piezoelectric harvesters. IEEE Access.

[37] Chew Z.J., Zhu M. (2020). Adaptive self-configurable rectifier for extended operating range of piezoelectric energy harvesting. IEEE Trans. Ind. Electron..

[38] Costanzo L., Lo Schiavo A., Vitelli M. (2023). A self-supplied power optimizer for piezoelectric energy harvesters operating under non-sinusoidal vibrations. Energies.

[39] Ben Ammar M., Sahnoun S., Fakhfakh A., Viehweger C., Kanoun O. (2023). Self-powered synchronized switching interface circuit for piezoelectric footstep energy harvesting. Sensors.

[40] Edla M., Lim Y.Y., Mikio D., Padilla R.V. (2022). Non-linear switching circuit for active voltage rectification and ripples reduction of piezoelectric energy harvesters. Energies.

[41] Haseeb A., Edla M., Ucgul M., Santoso F., Deguchi M. (2023). A voltage doubler boost converter circuit for piezoelectric energy harvesting systems,”. Energies.

[42] Haseeb A., Edla M., Thabet A.M., Deguchi M., Kamran M. (2023). A self-powered dual-stage boost converter circuit for piezoelectric energy harvesting systems. Energies.

[43] Kamran M., Edla M., Thabet A.M., Haseeb A., Mikio D., Bui V. (2023). A self-powered FBRJT AC-DC conversion circuit for piezoelectric energy harvesting systems. Energies.

[44] ADP5090 Ultralow Power Boost Regulator with MPPT and Charge Management—Datasheet. Analog Devices, https://www.analog.com/en/products/adp5090.html.

[45] Evaluation Board for the ADP5090 Ultralow Power Boost Regulator— EVAL-ADP5090. Analog Devices,. https://www.analog.com/media/en/technical-documentation/user-guides/EVAL-ADP5090_UG-708.pdf.

[46] M. Aleksandrova, I. Pandiev, Printed piezoelectric harvester for integration in a wearable energy storage device, 47^th^ International Spring Seminar on Electronics Technology, 15-19 May, Prague, Czech Republic.

[47] Aleksandrova M. (2020). Polymeric seed layer as a simple approach for nanostructuring of Ga-doped ZnO films for flexible piezoelectric energy harvesting. Microelectron. Eng..

[48] Say M.G., Brett C.J., Edberg J., Roth S.V., Söderberg L.D., Engquist I., Berggren M. (2022). Scalable paper supercapacitors for printed wearable electronics. ACS Appl. Mater. Interfaces.

[49] Yan Z., Luo S., Li Q., Wu Z.S., Liu S.F. (2024 Feb). Recent advances in flexible wearable supercapacitors: properties, fabrication, and applications. Adv. Sci..

